# Double inv(3)(q21q26.2) in acute myeloid leukemia is resulted from an acquired copy neutral loss of heterozygosity of chromosome 3q and associated with disease progression

**DOI:** 10.1186/s13039-015-0171-2

**Published:** 2015-08-19

**Authors:** Jun Gu, Keyur P. Patel, Bing Bai, Ching-Hua Liu, Guilin Tang, Hagop M. Kantarjian, Zhenya Tang, Ronald Abraham, Rajyalakshmi Luthra, L. Jeffrey Medeiros, Pei Lin, Xinyan Lu

**Affiliations:** Cytogenetic Technology Program, School of Health Professions, The University of Texas, MD Anderson Cancer Center, 1515 Holcombe Blvd. Unit 0002, Houston, TX 77030 USA; The Department of Hematopathology, The University of Texas, MD Anderson Cancer Center, 1515 Holcombe Blvd. Unit 0149, Houston, TX 77030 USA; Department of Leukemia, The University of Texas, MD Anderson Cancer Center, 1515 Holcombe Blvd. Unit 0428, Houston, TX 77030 USA

## Abstract

**Background:**

Acute myeloid leukemia (AML) with inv(3)(q21q26.2)/t(3;3)(q21;q26.2) is a distinct clinicopathologic entity with a poor prognosis. However, double inv(3)(q21q26.2) is extremely rare in AML. We report here 3 cases analyzed by oligonucleotide microarray comparative genomic hybridization (aCGH) and single nucleotide polymorphism (SNP). Clinicopathologic, cytogenetic and molecular findings were correlated with clinical outcome to better understand the entity.

**Results:**

The study group included one man and two women at 56–74 years of age. The AML arose from myelodysplastic syndrome in one patient and from chronic myelomonocytic leukemia in another patient. Monosomy 7 was found as additional cytogenetic finding in one patient. One patient had a single inv(3) in the initial clone and acquired double inv(3) as part of clonal evolution. *EVI1 (MECOM)* rearrangement was confirmed using metaphase/interphase fluorescence *in situ* hybridization (FISH). Microarray (aCGH + SNP) data analysis revealed that the double inv(3) was a result of acquiring copy neutral loss of heterozygosity of chromosome 3q: arr[hg19] 3q13.21q29(10,344,387–197,802,470)x2 hmz, spanning ~ 94.3 Mb in size. Mutational profiling showed a *PTPN11* mutation at a low level (~10 %) in one patient and wild type *FLT3* and *RAS* in all patients. No patients achieved cytogenetic remission and all died with an overall survival (OS) of 23, 12 and 5 months, respectively.

**Conclusions:**

Double inv(3) is a result of acquired copy neutral loss of heterozygosity, a somatic repair event occurring as a part of mitotic recombination of the partial chromosome 3q. The double inv(3) in AML patients is highly associated with a rapid disease progression.

## Background

The 2008 World Health Organization (WHO) classification recognized acute myeloid leukemia (AML) with inv(3)(q21q26.2) or t(3;3)(q21;q26.2) and *GATA1-EVI1(MECOM)* rearrangement as a clinicopathologic entity, associated with poor clinical outcomes. This disease accounts for less than 2 % of all cases of AML [1, 2] including *de novo* and AMLs transformed from myelodysplastic syndrome (MDS) [3]. This cytogenetic abnormality also can occur in blast phase of chronic myelogeneous leukemia (CML) [4]. *GATA1*-*MECOM*(*EVI1*) resulting from inv(3)/t(3;3) is known to play a critical role in the leukomogenesis and highly associated with other chromosomal aberrations such as monosomy 7 or 7q deletion (−7/7q-) or a complex karyotype, although these additional cytogenetic findings do not have independent prognostic value in this entity[5].

A double inv(3)(q21q26.2) occurs when the paracentric inv(3) involves both chromosome 3 homologues. Double inv(3)(q21q26.2) is an extremely rare event with about 10 cases reported in the literatures, primarily in AML patients [6–9] and very rarely in MDS [10] or the blast phase in CML [11, 12]. The underlying mechanism for double inv(3) and its clinical impact remains largely unknown.

Although double inv(3) can be detected by the traditional chromosome analysis and/or by fluorescence *in situ* hybridization (FISH) targeting the *MECOM/EVI1* gene locus, both techniques cannot delineate the potential underlying mechanism leading to this abnormality. Earlier studies postulated that the double inversion event could be due to loss of the normal chromosome 3 homolog followed by the duplication of the inverted abnormal chromosome 3 [6, 7]. One recent report using single nucleotide polymorphism (SNP) microarrays revealed evidence of an acquired copy neutral loss of heterozygosity (aCN-LOH) or acquired segmental uniparental disomy (aUPD) of only chromosome 3q, instead of the entire chromosome 3, in a CML patient in blast phase [11].

Single nucleotide polymorphism microarray based technology has clinical utility in the diagnosis and risk stratification of AML patients can identify clinically relevant copy number aberrations and importantly can detect acquired segmental aUPD or aCN-LOH in the tumor genome especially in those myeloid neoplasms with normal karyotypes [13, 14]. The aCN-LOH, resulting from the apparent duplication of oncogenic mutations coupled with the loss of the normal alleles, has been postulated to be associated with myeloid malignancies [15].

To better understand the clinical features as well as the potential underlying genomic events associated with the unique subgroup of double inv(3) in AML patients, we performed a retrospective data review and aCGH + SNP analysis. We also correlated the clinicopathologic, molecular and cytogenetic data with clinical outcome.

## Results

The study group included one man and two women who were 72, 64 and 56 years of age, respectively, at the diagnosis of AML. All demographic data are summarized in Tables 1 and 2.

**Table 1:** Summary of clinical data

Case no.	Patient 1	Patient 2	Patient 3
Age	72	64	56
Gender	Male	Female	Female
Initial referring diagnosis	MDS-RAEB-2 for <1 month	CMML <3 months	Pancytopenia/thrombocytopenia for one week
Final diagnosis at disease progression	AML	AMML	AML
WBC (× 10^9^/L)	2.4	8.9	1.6
Hb (g/dL)	8.5	11.8	8.2
Platelets (× 10^9^/L)	110	242	41
MCV (fl)	109	112	99
Neutrophil percent (%)	45	44	5.6
Lymphocyte percent (%)	50	22	90.8
Monocyte percent (%)	4	33	1.8
BM blasts %	28	23	54
Follow up (months)	4	3	3.5
Treatment	ara-C, imatinib vorinostat	decitabine	idarubicin, cytarabin
Complete Remission (CR)	no	no	morphological
Stem cell transplant	no	no	no
Overall survival (months)	23	12	5

**Table 2:** Summary of cytogenetic and molecular results

Case no.	Patient 1	Patient 2	Patient 3
Cytogenetics	46,XY,inv(3)(q21q26.2)[13]/46,idem,del(7)(q22)[1]/46,XY,inv(3)(q21q26.2)x2[12]/46,XY[7]	46,XX,inv(3)(q21q26.2)x2[18]/46,XX[2]	46,XX,inv(3)(q21q26.2)x2[1]/45,idem,-7 [14]/46,XX[5]
FISH	ND	ND	*MECOM/EVI1*
aCGH + SNP	aCN-LOH chr3q	ND	monosomy 7 (<10 %)
Molecular study			
*FLT3*	-	-	-
*K/N-RAS*	-	-	ND
*PTPN11*	ND	ND	+ missense mutation at a very low allelic frequency (<10 %)
*CEBPA*	ND	ND	+ Germline Variant

*aCN-LOH* acquired copy neutral loss of heterozygosity, *Chr3q* chromosome 3q, *ND* not done, “-” negative, “+” positive

### Patient 1

Patient 1 was a 72 year-old Hispanic man diagnosed with a myelodysplastic syndrome (refractory anemia with excess blasts-2) with ~15 % blast at a local hospital one month prior to his first visit to MDACC. The bone marrow was heypercellular and involved by acute myeloid leukemia with 28 % of blasts. Flow-cytometry immunophenotyping showed that the blasts were positive for CD13, CD33, CD34, CD38, CD117 and HLA-DR.

**Fig. 1:**
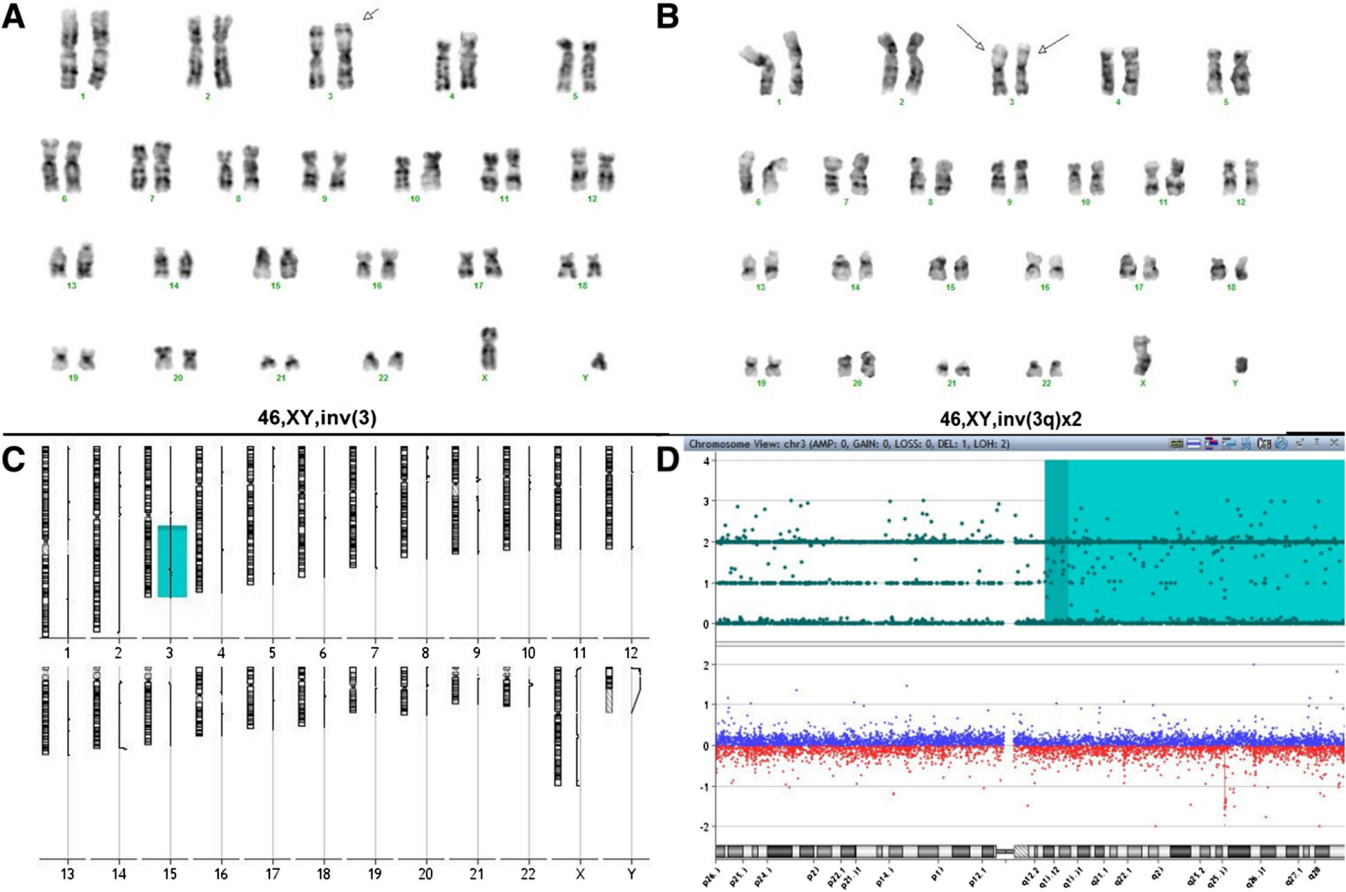
Karyotypes from patient **a** showing 46,XY,inv(3)(q21q26.2) at the diagnosis and **b** 46,XY,inv(3)(q21q26.2)x2 at disease progression. ACGH + SNP showed evidence of aCN-LOH of chromosome 3q **c** whole genome view and **d** chromosome 3 only with 3q highlighted in light blue

Cytogenetic analysis showed a single inv(3) (Fig. 1a) as a part of 46,XY,inv(3)(q21q26.2) [13]/46,idem,del(7)(q22)[1]/46,XY[7]. Molecular studies for *FLT3* and *K/N-RAS* were wild type. The patient was treated with reduced dose cytarabine and imatinib but did not respond and after two months of therapy the bone marrow showed 79 % blast. This coincided with cytogenetic evidence of evolution from single inv(3) to double inv(3) (Fig. 1b) in the following karyotype 46,XY,inv(3)(q21q26.2)[3]/46,XY,inv(3)(q21q26.2)x2[13]/46,XY[4]. The patient was switched to vorinostat (suberanilohydroxamic acid or SAHA) therapy due to refractory disease. Although his disease was stable for a short period of time clinically, he had persistent disease without achieving complete remission (CR). In the ensuing 4 months, the double inv(3) became predominant as the only abnormal clone. He died 23 months after initial diagnosis of AML.

Retrospective aCGH + SNP was performed on the bone marrow sample with double inv(3) and showed aCN-LOH of chromosome 3q:arr[hg19] 3q13.21q29(10,344,387–197,802,470)x2 hmz, spanning ~ 94.3 Mb in size (Fig. 1c and d). No additional clinically relevant copy number aberrations were observed at the level of the aCGH + SNP analysis applied.

### Patient 2

Patient 2 was a 64-year-old Caucasian woman who presented with monocytosis and suspected chronic myelomonocytic leukemia (CMML) type 2 three months prior to visiting our hospital. At our institution, bone marrow examination showed acute myelomonocytic leukemia with 23 % blasts. Flow-cytometry immunophenotyping showed the blasts were positive for CD13, CD14, CD15, CD33, CD38, CD64 (major subset), MPO and HLA-DR and negative for CD34, CD117.

Chromosome analysis and FISH targeting chromosomes 5/5q, 7/7q, 8 and 20q were all reported to be normal at the outside hospital. However, the first bone marrow at our hospital showed double inv(3) as predominant clone in a karyotype of 46,XX,inv(3)(q21q26.2)x2 [18]/46,XX[2]. Molecular studies showed that *FLT3* and *RAS* were wild type. The patient was treated with decitabine and showed no response. She was followed up for 3 months and the double inv(3) clone was a persistent finding . The patient died 12 months after initial diagnosis; aCGH + SNP was not performed due to unavailability of diagnostic materials.

### Patient 3

Patient 3 was a 56-year-old Caucasian woman with a history of pancytopenia and thrombocytopenia for one week prior to her visit at MDACC. Flow-cytometry immunophenotyping studies showed that the blast were positive for CD2 (partial), CD4 (partial), CD13, CD14 (partial), CD15 (partial), CD22 (partial), CD33, CD34, CD38, CD56 (partial), CD64 (partial), CD117 (partial), CD123 (dim)and HLA-DR (partial). The blasts were negative for CD3 (surface and cytoplasmic), CD5, CD7, CD10, CD19, and myeloperoxidase.

**Fig. 2:**
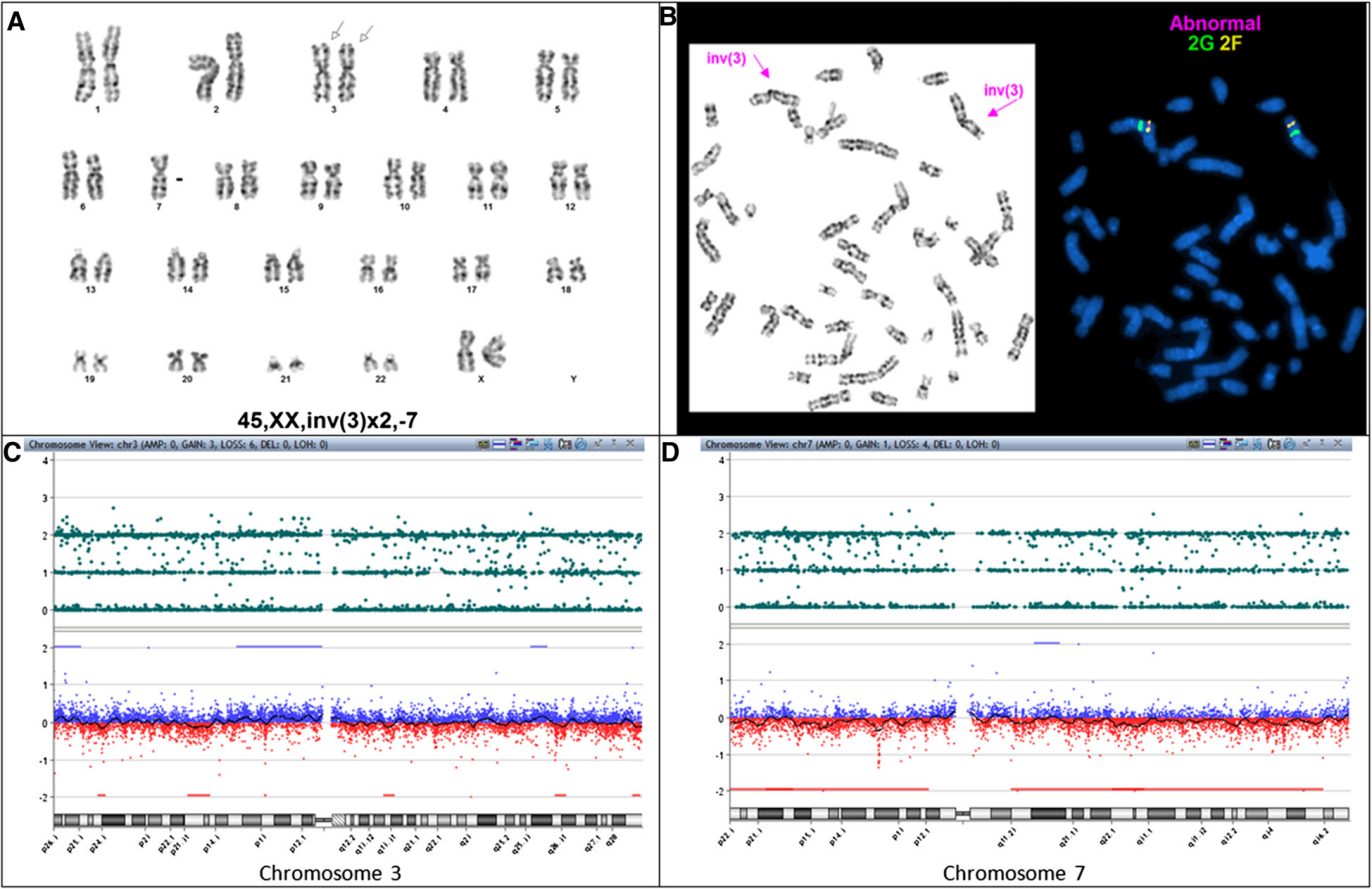
Karyotype from patient 3 showing **a** 45,XX,inv(3)(q21q26.2)x2,-7 and **b** a metaphase FISH study using *EVI1(MECOM)* showing breakapart of green and red signals on both chromosome 3. ACGH + SNP analysis of patient 3 showed no apparent evidence of CN-LOH on chromosome 3q (**c**) and a very low level of monosomy 7 with a black line slightly below the zero line (**d**)

Chromosome analysis showed double inv(3) as the primary clone with a large subset cells showing monosomy 7 (Fig. 2a) as additional finding in the secondary clone in a karyotype of 46,XX,inv(3)(q21q26.2)x2[3]/45,idem,-7[14]/46,XX[3]. Double *EVI1 (MECOM)* rearrangement was confirmed by metaphase FISH (Fig. 2b). *FLT3* and *RAS* were wild type. However, a next generation sequencing (NGS) targeting 28 genes on this patient showed a *PTPN11* missense mutation with ~ 10 % allelic frequency indicating a low level somatic event.

A retrospective aCGH + SNP was performed on the diagnostic bone marrow. However, microarray data showed no apparent indication of aCN-LOH on chromosome 3q (Fig. 2c) and a very low level (~10 %) of mosaicism of monosomy chromosome 7 (Fig. 2d). Although aCGH + SNP did not demonstrate the expected aCN-LOH, the low level mosaicism and clonality for monosomy 7 were consistent with *PTPN11* mutation. The discrepancy observed between cell based cytogenetic/FISH analysis and DNA based molecular and aCGH + SNP analyses, is most likely attributable to a low percentage of tumor DNA content in this patient’s specimen, possibly related to DNA degradation of the tumor cells or a high level of contaminated normal cells.

The patient received induction idarubicin and cytarabine and achieved a morphological complete remission (CR) on day 28, although cytogenetically the double inv(3) was persistent in 10 of 20 cells (50 %) and she died 5 months after initial diagnosis.

## Discussions

We report three new cases of AML with double inv(3). Although a small series, this is the largest report to date and we performed a comprehensive review of the clinicopathologic, molecular and cytogenetic data and correlated the findings with clinical outcome.

Double paracentric inv(3q) is a very rare event with less than 10 cases reported in myeloid neoplasms [6, 16–19]. All reported cases showed involvement of chromosomal bands (3q21 and 3q26.2). However, very limited clinical data were available on most of these cases and almost no molecular and cytogenomic data are available. To better understand this subset of the patients, we reviewed and summarized our data with those 10 cases from literatures in Table 3. Among 12 total cases, six were women and six were men, with an average age of 62 years (range 36–80) at diagnosis [6]. Most of the double inv(3) cases were classified as AMLs (*N* = 9, 75 %) with one case of MDS [10] and two cases were CML in blast phase (BP) [12, 11]. Monosomy 7 was the most common additional cytogenetic finding, observed in five cases (41.7 %) and 7q deletions were seen in two cases. In two CML cases with BP, the double inv(3) co-existed with the Ph clone or *BCR-ABL1* fusion [11]. Similar to AML patients with single inv(3) [2, 5], most patients with double inv(3) patients showed no or a minimal response to the conventional chemotherapy and rapid disease progression. All our patients were negative for *FLT3* or *RAS* mutations, indicating that the double inv(3) entity is less associated with these somatic mutations that have been frequently reported in AML.

**Table 3:** Summary of 12 cases with double inv(3)(q21q26.2) reported in the literature

Age (year)/gender	Blast (%)	Diagnosis	Cytogenetic findings	Overall survival (mo)	Reference
55/F	NA	MDS	46,XX,inv(3)x2	24	Walter [10]
NA/F	NA	BP-CML	46,XX,inv(3)x2,t(9;17;22)	NA	Levy [12]
80/M	NA	AML-M1	46,XY,inv(3)x2	13	Secker-Walker [8]
39/M	NA	AML-M4	45,XY,inv(3)x2,-7	3	Secker-Walker [8]
83/F	63	AML	45,XX,inv(3)x2,-7	NA	Lee [7]
65/M	49	AML-M4	46,XY,inv(3)x2,	24	Lahortiga [9]
36/M	NA	CML	46,XY,inv(3)x2,7q-	NA	Toydemir [11]
62/M	14	AML-M1	45,XY,inv(3)x2,-7	9	De Braekeleer [6]
67/F	35	AML	46,XX,inv(3),5q+/45,idem,-7/45,idem,inv(3),-7	4	De Braekeleer [6]
72/M	79	AML-M6A	46,XY,inv(3)(q21q26.2),del(7)(q22)/46,XY,inv(3)(q21q26.2)x2	23	Patient 1
64/F	23	AMML	46,XX,inv(3)x2	12	Patient 2
56/F	54	AML	46,XX,inv(3)x2/45,idem,-7	5	Patient 3

*NA* not available, *MDS* myelodysplastic syndrome, *AML* acute myeloid leukemia, *CML* chronic myelogeneous leukemia, *BP* blast phase, *AMML* acute myelomonocytic leukemia, *Mo* months

Overall survival (OS) data were available in 9 of 12 cases with the double inv(3) (Table 3). Compare with a previously reported median overall survival of 8.9 months in single inv(3) or t(3;3) positive AML cases [5], the median OS for the double inv(3) AML cases was 12 months (range, 3–24 months). This comparison might indicate that double inv(3) does not impose an extra risk on the OS, however, we note that patients with concurrent additional monosomy 7 (*N* = 4) seemed do worse (Table 3) and showed a median OS of 4.5 months (range 3–9 months) compared with patients without monosomy 7 (*N* = 5), who had a median OS of 23 months (range 12–24 months) (*p* = 0.004). Although monosomy 7 did not show independent prognostic value in single inv(3) AML patients [5], it apparently has adverse impact on AML patients with the double inv(3).

There are three possible mechanisms of double inv(3q) formation. The first proposed mechanism is that double inv(3) is the result of loss of the normal chromosome 3 followed by a somatic rescue event resulting in duplication of the inverted chromosome 3 [10]. This mechanism would result in uniparental disomy (UPD) for the entire chromosome 3 which was not consistent with what we have observed in patient I in this study who showed segmental aCN-LOH at the chromosome 3q13.21q29. The second proposed mechanism is that double inv(3) could be due to a somatic repair resulting from nonallelic homologous recombination (NAHR) or nonhomologous end-joining (NHEJ) [20] or in short, a somatic recombination event in cancer genome. This mechanism seems more consistent with our aCGH + SNP findings of chromosome 3q: arr[hg19] 3q13.21q29 (10,344,387–197,802,470)x2 hmz, which was almost identical to what has been reported in a CML patient in blast phase with double inv(3) [11], by a high-resolution SNP microarray analysis. The SNP array data in these two double inv(3) patients (our patient 1 and the CML patient) suggest that the double inv(3) was a result of somatic rescue with loss of the chromosome 3 and coupling with a subsequently partial duplication of 3q [16, 11, 18], or a result of a mitotic recombination of the chromosome 3q. The third mechanism proposed is that the double inv(3q) could occur on both chromosome 3 homologues independently although it is less likely and no studies have ever provided evidence to support this hypothesis.

Microarray based testing including both SNP microarray or oligonucleotide aCGH + SNP is a powerful molecular cytogenetic tool for detecting aberrations that are cytogenetically undetectable but clinically relevant. These methods have been fully utilized in the clinical diagnosis of constitutional disorders [21] and also are widely implemented in cancer genetics, particularly in myeloid neoplasms [22]. However, it is important to note that microarray based testing relies on the level of mosaicism or clonality in cancer diagnosis. False negative array results such as observed in our patient 3 can occur if the tumor sample tested is compromised by high level normal cell contamination.

The CN-LOH or UPD can only be detected by molecular methods or SNP based microarray testing. Acquired CN-LOH or UPD (aCN-LOH or aUPD) have been frequently reported in myeloid neoplasms [15, 23]. Some aCN-LOH regions encoding known oncogenic mutations such as *JAK2, MPL, c-KIT, FLT3, RUNX1* on chromosomes 9p24.1, 1p34, 4q21, 13q12 and 21q22, respectively, are frequently recurrent in AML and MDS, resulting in a “double hit” or “homozygous mutations”. Recent studies have shown that these homozygous mutations are the results of clonal evolution [24] in AML. As observed in patient 1, cytogenetically the double inv(3) evolved from the single inv(3) during the disease progression confirming that the aCN-LOH is the result of clonal evolution.

## Conclusions

We reported three new AML cases with the double inv(3) with comprehensive analysis of clinicopathologic, cytogenetic and molecular/genomic data and patient clinical outcome. We also compared our data with what has been reported in the literatures. We conclude that double inv(3) results from aCN-LOH, a somatic repair event of mitotic recombination of the partial chromosome 3q. The double inv(3) is frequently associated with monosomy 7 and should be recognized as a unique cytogenetic entity in myeloid neoplasms especially in AML patients. This subset of patients often shows a rapid disease progression with a dismal OS.

## Methods

### Patient inclusion

This study was approved by the institutional review board at The University of Texas MD Anderson Cancer Center (MDACC). Retrospective cytogenetic data review was performed on all patients diagnosed with myeloid neoplasms tested in the Clinical Cytogenetics laboratory between 2002 and 2014 at MDACC. Only patients with double inv(3) were included to the study.

### Morphologic and flow-cytometry immunophenotypic analyses

Hematoxylin-eosin-stained histologic sections of bone marrow biopsy specimens, and Wright-Giemsa stained peripheral blood and bone marrow aspirate smears were reviewed. Complete blood cell and differential counts were performed on peripheral blood smears of all three patients.

Four- or eight- color flow cytometry immunophenotypic analysis was performed as described according to standard procedures. The panel included antibodies directed against: CD3, CD4, CD5, CD7, CD9, CD10, CD13, CD19, CD20, CD22, CD25, CD33, CD34, CD38, CD52, CD79a, CD117, BCL-2, HLA-DR, TdT, myeloperoxidase, IgM (cytoplasmic), kappa and lambda light chains. All antibodies were obtained from Becton-Dickinson Biosciences (San Jose, CA, USA), except for TdT (Supertechs Inc, Bethesda, MD, USA).

### Cytogenetic and FISH analysis

Twenty-four and/or forty-eight hour unstimulated bone marrow cultures were setup for chromosome preparation, following standard chromosome harvesting procedure. Twenty metaphases were analyzed using a Leica-microscope imaging system (Leica Microsystems Inc., Chicago, IL) and karyotypes were described according to International System for Human Cytogenetic Nomenclature (ISCN 2009 and 2013).

FISH for *EVI1 (MECOM)* rearrangement was performed on cultured bone marrow metaphases and interphases, using a breakapart probe from Kreatech (Leica Microsystems Inc. Chicago, IL) according to the manufacturer’s instructions.

### Oligonucleotide microarray comparative genomic hybridization + single nucleotide polymorphism (aCGH + SNP)

Genomic DNA was extracted from bone marrow aspirate material using Gentra Puregene (Qiagen, Valencia, CA). The Cancer Cytogenomic Microarray Consortium (CCMC) 4x180K chip was used according to the manufacturer’s protocol (Agilent Technologies, Santa Clara, CA) as described previously [25]. The Agilent GeneChip microarray scanner was used for imaging and data analysis was performed using Cytogenomic workbench software.

### Molecular study

Genomic DNA was PCR amplified and subjected to mutation analysis for codons 12, 13 and 61 of *KRAS* and *NRAS* by pyrosequencing using a PSQ HS 96 Pyrosequencer (Biotage, Uppsala, Sweden). CEBPA was assessed by direct Sanger sequencing on an ABI Prism 3100 Genetic Analyzer (Applied Biosystems, Foster City, CA) [26]. A fluorescence-based multiplex PCR was used to detect internal tandem duplication (ITD) and D835 point mutation of the *FLT3* gene. The PCR products were then subjected to capillary electrophoresis on an ABI Prism 3100 Genetic analyzer to distinguish wild and mutant genotypes. For the third patient *KIT, NPM1, KRAS* and NRAS mutation were assessed using a next generation sequencing (NGS)-based assay [27].

## Consent

Informed consent was waived per institution IRB.
